# Can evolutionary immunology decode micro and nanoplastic challenges?

**DOI:** 10.3389/fimmu.2024.1404360

**Published:** 2024-05-17

**Authors:** Andi Alijagic, Eva Särndahl

**Affiliations:** ^1^ Man-Technology-Environment Research Center (MTM), Örebro University, Örebro, Sweden; ^2^ Inflammatory Response and Infection Susceptibility Centre (iRiSC), Örebro University, Örebro, Sweden; ^3^ School of Medical Sciences, Faculty of Medicine and Health, Örebro University, Örebro, Sweden

**Keywords:** innate immunity, particles, environmental species, humans, pattern recognition receptors (PRRs)

## Introduction

The exceptional versatility and broad applicability of plastics in various industries and daily life is resulting in escalating release rates of plastics across environments. Under varying environmental conditions, plastics undergo fragmentation into so-called microplastic particles (<5 mm) and nanoplastic particles (≤100 nm) with increased risks for exposure and scarcely explored hazardous effects on humans and environmental species (1–3). Considering their small size, micro and nanoplastic particles (MNPs) are expected to interact with the epithelial barriers in, for example, gills, lungs, digestive tract, or exoskeleton of various species. Such interactions may result in receptor-dependent or -independent modulation of cellular signaling machinery maintaining the overall function and homeostasis of the cell. As the essential protective system defending the organism’s integrity and well-being, the innate immune system’s structures and mechanisms stand as the first pillar in the recognition and elimination of foreign materials and potential threats, including MNPs. Scientific efforts to explore MNPs-innate immune interactions are well undergoing, however, mainly by separate studies with the focus on individual environmental species, ranging from bivalves and earthworms to echinoderms and vertebrates. In order to grasp the full picture of the MNPs-innate immune interactions and understand their outcomes, leveraging the evolutionary immunology approach offers numerous advantages. Critically, a broad-taxa approach covering different phyla and hierarchical levels of organization is an important concept in (eco)toxicological studies and pollution monitoring (4) and provides significant potential in tackling MNP challenges.

## Tracing the innate immunity across phyla and species

**Figure 1:**
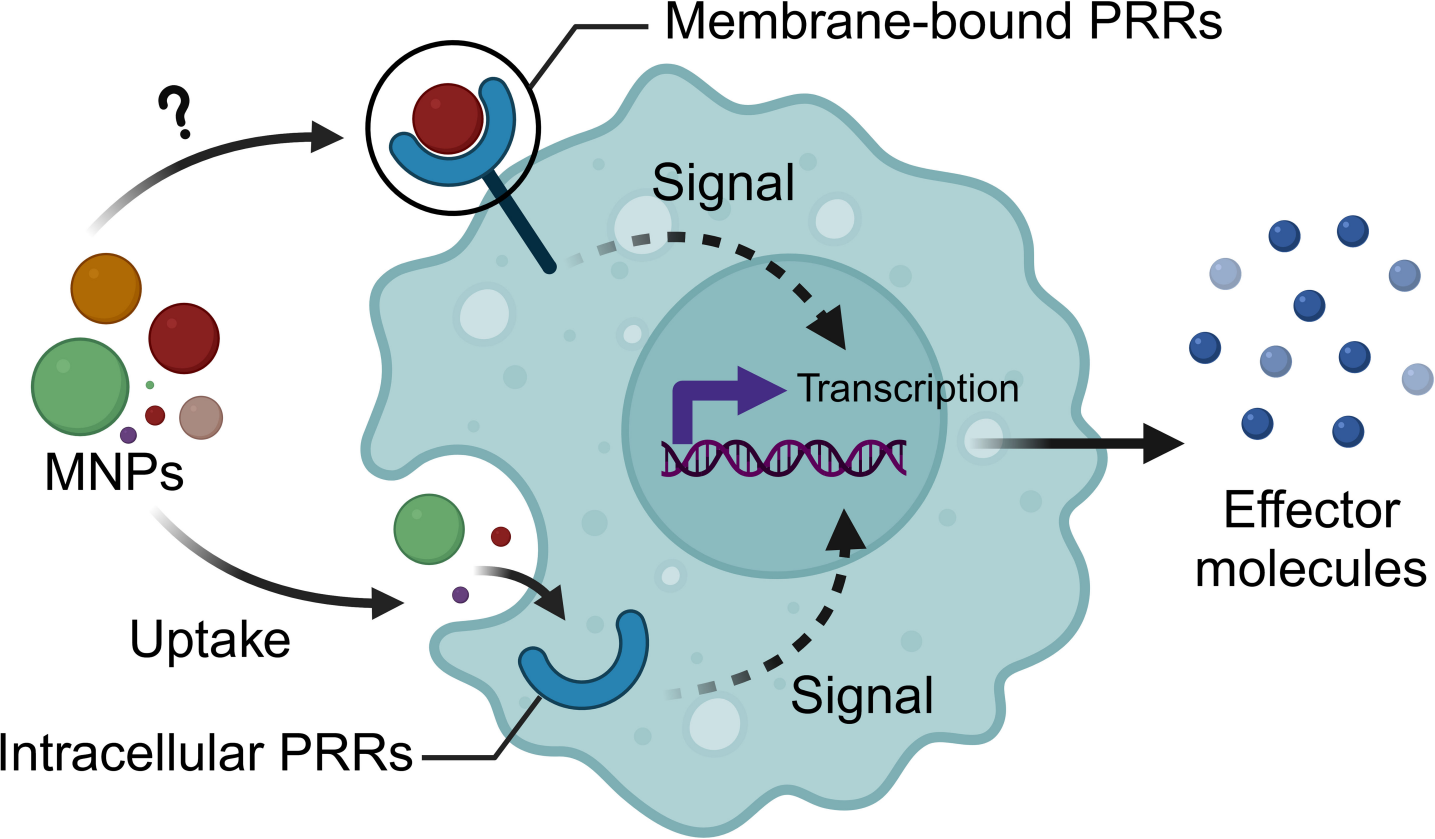
The general traits of the innate immune system found in phagocytic cells across species.

The innate immune system holds several molecular and structural traits that are conserved throughout evolution, as summarized in **Figure 1**. Those include subsets of circulating or tissue-resident innate effector cells and an intricate network of receptors (e.g., pattern recognition receptors - PRRs) and signaling cascades (5). Cross-species effector functions of innate cells predominantly rely on phagocytosis combined with the generation of reactive oxygen or nitrogen species (ROS/NOS), production and release of lysosomal enzymes, antimicrobial peptides, cytokines, fibrinogen-related peptides, leucine-rich repeats (LRRs), and complement-related proteins. Additional innate effector functions described across phyla are agglutination, clotting, coagulation, and melanization (4). Among PRRs, Toll-like receptors (TLRs) are present in all multicellular organisms and are typically expressed on sentinel cells, such as phagocytes, and have been recognized as key regulators of the immune responses. The prototypical TLR gene emerged in the eumetazoan ancestor approximately 600 million years ago, subsequently undergoing selection throughout evolution, likely to accommodate coevolving microbial threat cues. Genes involved in TLR-signaling pathways are highly conserved across both protostomes and deuterostomes and include the presence of TIR domain-containing adaptors, signaling mediators, effector molecules, and transcription factors activated by pathogens (6, 7). This compelling evidence speaks in favor of using innate immune response as a focal point in understanding common and distinct mechanisms and pathways modulated by MNPs, and in identifying predictive cross-species biomarkers of MNP exposure. Nevertheless, PRRs, including TLRs, exhibit genetic diversity, and to find gene homology between different metazoan species is challenging (8–10). This divergence in homology may lead to distinct functions between species (8–10), a factor that needs consideration when employing a cross-species approach in assessing MNP-innate interactions.

## Lessons learned from engineered nanomaterials and air pollution particles

Based on the existing knowledge about cross-species immunosafety assessment of engineered nanomaterials (ENMs) or air pollution particles, there are many lessons learned that could be applied in the immunosafety assessment of MNPs. It is assumed that MNPs follow similar mechanisms of action as ENMs. Consequently, they ought to have the capacity to trigger the activation of innate immune mechanisms ranging from recognition to internalization and inflammatory response (6). However, when evaluating such interactions, it is of crucial importance to consider several aspects including physicochemical properties of particles, for example, size, shape, intrinsic and surface chemistry, aggregation/agglomeration affinity, etc. (11). Importantly, ENMs readily interact with biomolecules present in the environment and/or biological systems and form biomolecular interface or (eco)corona that is largely shaping innate immune responses (12, 13). Similar concepts are also being applied in the case of MNPs (14). Notably, studies on potential recognition of MNPs by surface and intracellular PRRs are still in their early stages. Currently, these studies primarily provide evidence of increased protein levels or gene expression of PRRs or downstream signaling molecules following MNP exposure, rather than direct evidence of receptor binding (15–17). Moreover, when defining the immune responses to MNPs, and as learned for ENMs, it is of paramount importance to clearly distinguish between physiological and anomalous innate response. Inflammatory response *per se* is considered a physiological defensive response across species. However, failure of the inflammatory resolution or defective response may result in pathological consequences for the organism (12).

## Interweaving evolutionary immunology with micro and nanoplastic challenges

Based on the extensive body of evidence, studying the MNP effects on the innate immune system in the evolutionary immunology context would yield manifold benefits. Primarily, it would provide important insight into the common and distinct mechanisms that innate immunity, as a first and conserved line of defense, employs to cope with MNP particles, from basal eukaryotic organisms to humans. Moreover, different species occupy different trophic levels and ecosystems, and understanding MNPs-innate interactions may provide essential insights on how MNP exposure affect species lower down the food chain, or how MNPs move through the food chains and potentially impact human health. Species with high commercial value (e.g., mussels, crustaceans, sea urchins) as well as those with a crucial role in ecosystems (e.g., benthic organisms, fish, seabirds) are potentially exposed to MNPs. Hence, discerning the effects of MNPs on the innate immune system of these species could help in designing mitigation measures and regulatory actions in order to preserve their survival and biodiversity. Ecosystems are interconnected, and immunological and health changes in one species can have cascading effects on others. Studying the impacts of MNPs of innate reactivity across different species may further approach these complex interactions. The innate immune system of some species is more susceptible toward MNPs (18), and these species could serve as early-warning sentinels of MNPs as an urging pollution problem that is here to stay. On the contrary, some species have a more robust innate immune system [e.g., echinoderms have highly diversified panel of PRRs and effector molecules (19)] that could provide a base for understanding the mechanisms that help them cope with MNP pollution. In addition, cross-species hazard identification will enhance reliability when strategies for MNPs are set based on their physical and/or chemical properties. In addition, wide and multi-species understanding of MNPs-innate interactions could serve as an excellent platform to develop predictive bioassays, empowered with omics and high-throughput techniques, that will have the potential to replace, reduce, and refine (3R) animal use in research, mirroring the efforts with ENMs (4, 20).

## The future strategies for exploring MNP-innate immune interactions

Even though the cross-species approach in exploring MNP-innate immune system interactions holds promise, it faces several challenges. Firstly, it is highly important to establish standardized experimental models, both *in vitro* and *in vivo*, that simulate realistic exposure conditions across species, while also considering MNP physicochemical properties and environmental conditions affecting MNP behavior and interactions with the innate immune system. Further strategies should prioritize the development of *in vitro* models derived from key innate immune cell types, such as phagocytes, to investigate MNP-innate interactions from a mode of action perspective. Notably, mechanistic studies need to be designed feasibly, focusing on certain PRRs found in various species to allow for comparison. One example could be TLRs, the best-known PRRs present in all multicellular organisms, demonstrated to be promising in capturing cross-species effects of ENMs (5). Apart from focusing on individual molecules/pathways, additional endpoints such as phagocytic activity, cytokine/chemokine profiling, oxidative stress markers, and cell morphology should also be included. To evaluate the genetic determinants of MNP-innate interactions, genome-wide association studies and multi-omics analyses can identify genes, metabolites, and proteins involved in modulating innate immune responses to MNPs. Such an approach can identify gene candidates and target pathways, with further research scrutinizing their roles in MNP-innate interactions. Complementary broad-taxa *in vivo* models can validate *in vitro* findings and provide insights into systemic effects and organismal responses to MNP exposure. Longitudinal studies assessing chronic effects of MNP exposure on innate immune system functioning can provide important insights into the persistence of MNP-triggered effects on the innate immune system. Finally, integrating ecologically relevant endpoints, such as effects on species abundance and community structure, can serve as a connection between laboratory findings and real-life consequences of MNP pollution.

## Conclusions

The innate immune system, from basal eukaryotes to humans, holds several functional and structural characteristics that are conserved throughout evolution. The proper functioning of the innate immune system plays a crucial role in the survival and overall fitness of organisms. Therefore, in this opinion article, we emphasize the importance of adopting an evolutionary immunology framework to unveil the interactions between MNPs and the innate immune system across diverse species. For instance, assessing how invertebrate innate immune system responds to MNPs may reveal novel endpoints worth exploring in vertebrates and vice versa. Additionally, a broad-taxa approach may identify common innate mechanisms that transcend species boundaries. Leveraging evolutionarily conserved pathways and mechanisms identified in other organisms may be useful in translating findings to human health. Importantly, this broad-taxa approach aligns with the One Health initiative, which underscores the interconnectedness of human, animal, and environmental health. Furthermore, the proposed approach seeks to consolidate fragmented efforts and knowledge in exploring MNP-innate immune interactions in the time of escalating plastic pollution. We believe that the proposed evolutionary immunology approach, rooted in interdisciplinary research, will pave the way to a more accurate and reliable understanding of MNP-associated environmental and human health risks and informed decision making.

## Author contributions

AA: Conceptualization, Formal Analysis, Funding acquisition, Investigation, Project administration, Software, Writing – original draft, Writing – review & editing. ES: Funding acquisition, Project administration, Writing – original draft, Writing – review & editing.
